# Flotillin-1 downregulates K^+^ current by directly coupling with Kv2.1 subunit

**DOI:** 10.1007/s13238-016-0276-3

**Published:** 2016-05-24

**Authors:** Rui Liu, Guang Yang, Meng-Hua Zhou, Yu He, Yan-Ai Mei, Yu Ding

**Affiliations:** School of Life Sciences, Institutes of Brain Science and State Key Laboratory of Medical Neurobiology, Fudan University, Shanghai, 200438 China

**Dear Editor**,

Flotillin-1 (Flot1, reggie-2) is a lipid raft-associated scaffolding protein that belongs to a family of proteins characterized by an evolutionarily conserved SPFH domain (Browman et al., 2007; Langhorst et al., 2008). Although flotillin-1 is ubiquitously expressed, it is known to be highly expressed in brain tissue, and has been shown to serve distinct functions in the nervous system. Flotillin-1 has been shown to be necessary for axonal regeneration of retinal ganglion cells (Munderloh et al., 2009) and the differentiation and outgrowth of hippocampal neurons while additionally promoting the formation of glutamatergic synapses in the hippocampus (Swanwick et al., 2010). Recently, flotillin-1 has been identified as an evolutionarily-conserved memory related protein, involved in learning and memory processes (Monje et al., 2013). However, the ability of flotillin-1 to modulate critically important ion channel proteins, which are the most highly expressed membrane protein in brain, has not been previously demonstrated.

Voltage-gated potassium channels (Kv) are a member of the potassium channel family, which are all six transmembrane helices. There are 12 subfamilies, encoded by genes Kv1 to Kv11 (Yu et al., 2005). Delayed rectifier outward potassium channel (*I*_K_) is one of the most ubiquitously expressed voltage-gated K^+^ channels and plays many diverse physiological roles, due to its large conductance and slow inactivation properties. Besides setting the resting membrane potential and determining repolarization of action potential, our previous studies showed that enhancement of *I*_K_ is associated with the apoptosis, migration and maturation of neurons, depending on whether the neuron is in a normal development state or under abnormal apoptosis stimulation (Zhuang et al., 2012). Biochemical and functional evidence indicate that Kv2.1, the main α-subunit of *I*_K_ channels in neurons, localizes to non-caveolar lipid rafts (Martens et al., 2004), suggesting that the planar form lipid rafts which contain the flotillin-1 may be associated. Thus, it is interesting to investigate whether there was interaction between flotillin-1 and *I*_K_ channel or Kv2.1 protein, and if there was interaction, what’s the biological significance of their interaction.

We first overexpressed flotillin-1 in CGNs to address whether flotillin-1 overexpression would alter the amplitude of *I*_K_, or the gating properties of the channels. Up-regulating the expression of flotillin-1 in CGNs significantly reduced the *I*_K_ amplitude by 61.82% ± 5.94% (current amplitude from 1100.44 ± 56.64 pA reduced to 420.20 ± 65.32 pA, *n* = 20 and 9, *P* < 0.05) (Fig. S1A). The *G*-*V* curve analysis revealed no difference in the half-maximal activation voltage of *I*_K_ (Fig. S1B) between the control (31.24 ± 3.86 mV, *n* = 17) and flotillin-1 overexpressing neurons (32.56 ± 1.48 mV, *n* = 23). To study steady-state inactivation of *I*_K_ channels, we obtained an inactivation curve for *I*_K_ and calculated Vh_50_ (Fig. S1C). The half-maximal inactivation voltage of *I*_K_ currents in control neurons (13.6 ± 4.6 mV, *n* = 5) was similar to that in flotillin-1 overexpressing neurons (20.1 ± 2.1 mV, *n* = 3). These results suggest that the flotillin-1-mediated inhibition of *I*_K_ amplitudes cannot be attributed to a modification of *I*_K_ activation or inactivation properties.

In order to further elucidate the mechanism by which flotillin-1 affects Kv2.1 and the corresponding *I*_K_, we used HEK-293 cells as a model in the next set of experiments, because HEK-293 cells are known to be a suitable system for studying Kv2.1, due to the fact that they retain Kv2.1 neuronal expression pattern well (O’Connell et al., 2010). We also performed patch clamp recordings using HEK-293 cells, and obtained similar current recordings to those seen with CGNs (Fig. 1A and 1B). When flotillin-1 was overexpressed in HEK-293 cells co-transfected with Kv2.1, the Kv2.1 current amplitude was significantly reduced by 18.34% ± 7.03% (current amplitude from 4513.15 ± 375.66 pA reduced to 3627.35 ± 320.86 pA, *n* = 14 and 13, *P* < 0.05). By contrast, knocking down flotillin-1 expression via shRNA treatment in HEK-293 cells co-expressing Kv2.1 (Fig. S2), led to an increase in Kv2.1 current amplitude of 35.56% ± 13.50% (current amplitude from 3006.68 ± 355.88 pA enhanced to 4126.20 ± 421.80 pA, *n* = 13 and 13, *P* < 0.05). In addition, the steady-state activation and inactivation properties of Kv2.1 channels, following overexpression or knockdown of flotillin-1 in HEK-293 cells were similar to those observed from CGNs. Flotillin-1 overexpression or knockdown did not alter the half-maximal activation voltage of Kv2.1 current (Fig. 1C), with no significant difference seen between the control (31.0 ± 2.4 mV, *n* = 8), flotillin-1-overexpressing HEK-293 cells (22.3 ± 2.3 mV, *n* = 5) or shRNA knockdown cells (27.6 ± 2.2 mV, *n* = 6). Similarly, the half-maximal inactivation voltage of *I*_K_ currents in control cells (−20.2 ± 0.9 mV, *n* = 13) was similar to that seen in flotillin-1 overexpressing HEK-293 cells (−16.9 ± 1.0 mV, *n* = 14) or shRNA knockdown cells (−24.5 ± 1.7 mV, *n* = 8) (Fig. 1D).

**Figure 1:**
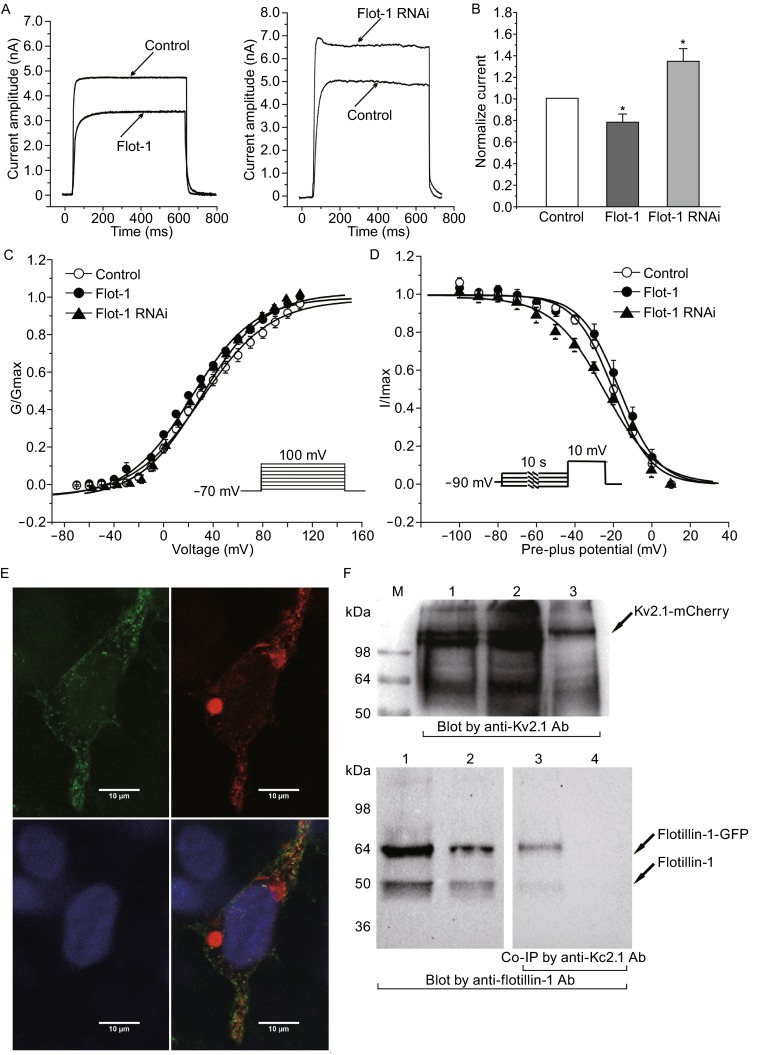
**The effects of flotillin-1 on Kv2.1 current amplitude, steady-state activation, steady-state inactivation properties and their co-localization in HEK-293 cells**. (A) Kv2.1 current evoked by a 200 ms depolarizing pulse from a holding potential from −50 to +40 mV. Current traces were obtained from HEK-293 cells co-transfected with Kv2.1 tagged with eGFP (Control), Kv2.1 and flotillin-1 (Flot-1) or Kv2.1 and flotillin-1 siRNA vectors (Flot-1 RNAi). (B) Statistical analysis of the effect of overexpression or knockdown of flotillin-1 on Kv2.1 current amplitude. (C) Steady-state activation curves of Kv2.1 current obtained from control, flotillin-1 overexpressing and flotillin-1 knockdown cells. Data points were fitted using the Boltzmann function. Data points are shown as mean ± SEM. (D) Steady-state inactivation curves of Kv2.1 current obtained from control, flotillin-1 overexpressing and flotillin-1 knockdown cells. (E) Immunofluorescent imaging showing co-localization between Kv2.1 and flotilin-1. Kv2.1 α-subunit was transfected into HEK-293 with a fluorescent mCherry tag (red channel), flotillin-1 was transfected with an eGFP fluorescent tag (green channel), DAPI was used to visualize the cell nuclei. Scale bar =10 μm. (F) The co-IP experiments show a direct interaction between Kv2.1 and flotillin-1. Upper, the co-IP using anti- Kv2.1 antibody linked to dynabeads protein G, blotted by Kv2.1 antibody; lower, blotted by anti flotillin-1 antibody. Lane 1, the supernatant of the lyzed cells, lane 2, the flow through fraction of co-IP, lane 3, the eluted fraction of co-IP, lane 4, negative control of co-IP

We then performed fluorescent imaging studies to determine whether Kv2.1 colocalizes with flotillin-1 in HEK-293 cells. The Kv2.1 α-subunit was transfected into HEK-293 cells with a mCherry marker, while the flotillin-1 plasmid contained an eGFP tag. Fluorescent imaging of these cells clearly show co-localization between Kv2.1 and flotilin-1 (Fig. 1E). Furthermore, co-IP experiments showed that a direct interaction between Kv2.1 and flotillin-1 could be detected (Fig. 1F).

In order to further characterize the interactions between flotillin-1 and Kv2.1, we employed the minimally invasive BiFC technique to directly visualize the protein complex formation in live HEK-293 cells (Fig. 2A). We first validated that the potential interaction between flotillin-1 and Kv2.1 is both physiologically relevant and not the result of over-expression (Fig. S3). The negative control NsfGFP and CsfGFP constructs showed that the background fluorescence was negligible (Fig. 2B). Since flotillin-1 and flotillin-2 were reported to form the hetero oligomer (Babuke et al., 2009), we constructed a pair of positive control BiFC constructs, consisting of flotillin-1-NsfGFP and flotillin-2-CsfGFP, and co-transfected them into HEK-293 cells. Figure. 2C shows that the formation of flotillin-1 and flotillin-2 hetero-oligomers activates the sfGFP BiFC complex, as evidenced by the green fluorescent clusters seen on the cell membrane. The distribution pattern of flotillin-1 and flotillin-2 formed a BiFC complex comparable to previous biochemical assay results obtained by other groups. We therefore studied the flotillin-1 and Kv2.1 interactions following similar protocol. The live HEK-293 cells co-expressing flotillin-1-NsfGFP and Kv2.1-CsfGFP exhibited high green fluorescence (Fig. 2D). The subcellular localization of flotillin-1 and Kv2.1 complex is clustered within a specific region of the cell membrane. Thus, we believe that flotillin-1 and Kv2.1 form a complex on the lipid rafts regions of the cell membrane *in vivo*.

**Figure 2:**
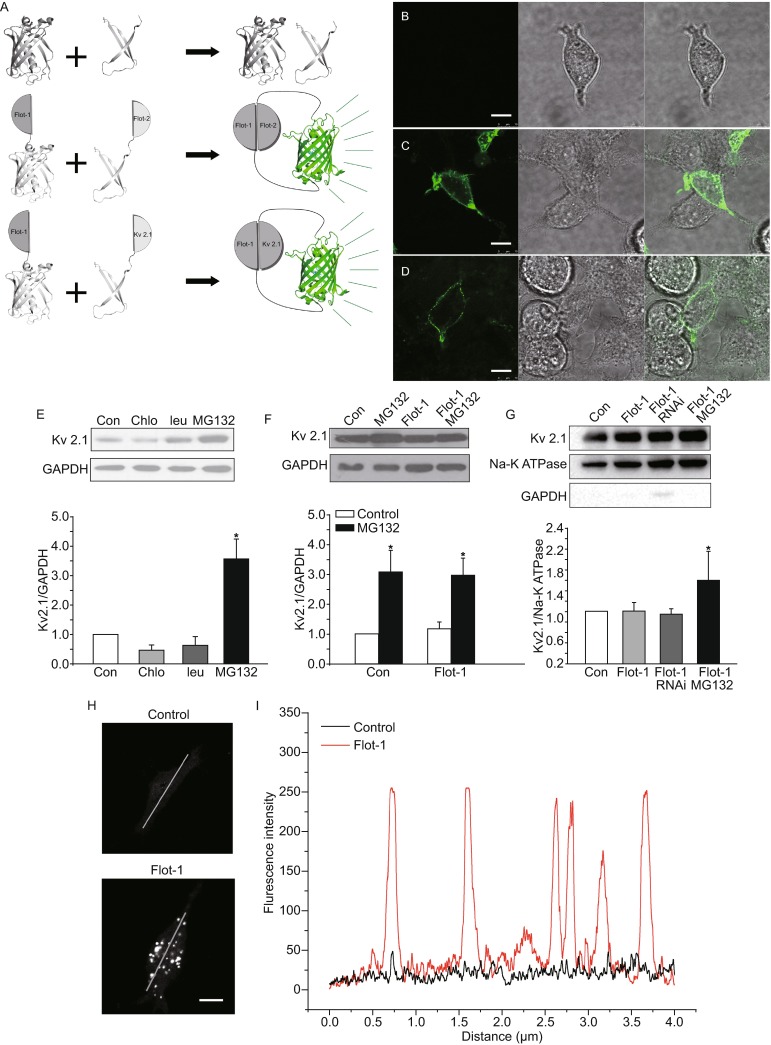
**Verifying the interaction between flotillin-1 and Kv2.1 by BiFC, Western blot and line scans analysis.** (A) Schematic diagram of the BiFC principle based on superfolder GFP (sfGFP). Co-transfection and expression of NsfGFP and CsfGFP fragment will not form the fluorescence complex (upper, grey). When NsfGFP and CsfGFP of sfGFP are fused to the target interaction proteins (Flot1 and Flot2/Kv2.1), if the two target proteins interact, this will facilitate the association between the NsfGFP and CsfGFP, that produces a bimolecular fluorescence complementation (BiFC) complex that can be observed *in vivo* (middle and lower, green). (B) The confocal image of living HEK-293 cells co-transfected with NsfGFP and CsfGFP without the fusion of additional proteins gave no detectable sfGFP fluorescence after 24 h transfection. (Left panel, green channel fluorescent image, middle panel, bright field image, right panel, overlay of the two images.) (C) The co-transfection of positive control Flot1-NsfGFP and Flot2-CsfGFP gave strong fluorescence in the cell membrane region. (D) The co-transfection of Flot1-NsfGFP and Kv2.1-CsfGFP give strong fluorescence at the cell membrane region. Scale bar = 10 μm. (E) Representative Western blots and statistical analysis for the effect of chloroquine (Chlo), leupeptin (leu) and MG132 on Kv2.1 expression levels (*n* = 4). Upper panels show representative images; lower panels show the densitometric analysis. Data are shown as mean ± SEM. *Denotes significant differences, with *P* < 0.05 compared with the corresponding control. (F) The effect of flotillin-1 on the total Kv2.1 expression level in the presence and absence of MG132 (*n* = 7). (G) Immunoblots and densitometric analysis of Kv2.1 expression on membrane surface, with either flotillin-1 knockdown or overexpression of flotillin-1 in the presence or absence of MG132. Proteins were isolated by the biotinylation assay. Membrane bound Na-K ATPase was used as the loading control. Endogenous GAPDH is shown as a cytoplasmic protein control. (H) Immunofluorescence imaging showing the co-expression of flotillin-1 resulted in increased Kv2.1 clustering on the membrane. (I) Line scans analysis of the distribution of Kv2.1 on the membrane. Plot of the fluorescence intensity (Y-axis) versus the respective location (X-axis) is shown. The white solid line in Fig. 2H indicates the 600 pixel segment used for the line scan analysis in Fig. 2I. Scale bar =15 μm

Previous studies reported that flotillin-1 can modulate the expression of cell surface receptors on the membrane by affecting their degradation process (Pust et al., 2013). It is known that proteins can be degraded via a lysosomal pathway or an ubiquitin proteasome pathway, and that the lysosomes likely degrade most membranes and endocytosed proteins. We thus explored whether the flotillin-1 mediated reduction in Kv2.1 current amplitude could be attributed to their role on Kv2.1 protein degradation. We first observed the main pathway of Kv2.1 protein degradation using the inhibitors of lysosomal or ubiquitin-proteasome pathway. Western blotting results show that the use of a lysosomal inhibitor (10 μmol/L chloroquine or 10 μmol/L leupeptin) did not significantly affect total Kv2.1 protein levels (Fig. 2E). By contrast, incubation of HEK-293 cells with a proteasome inhibitor (10 μmol/L MG132) led to a significant increase in Kv2.1 protein levels to 357.49% ± 68.02% of control (Fig. 2E). These results suggest that Kv2.1 is degraded mainly through the proteasome pathway. However, overexpression of flotillin-1 did not result in an increase of Kv2.1 degradation. As shown in Figure. 2F, with the application of MG132, the total protein levels of Kv2.1 in the presence and absence of flotillin-1 are not significantly different (*n* = 4, *P* > 0.05). These results suggest that although Kv2.1 is mainly degraded through the proteasome pathway, the inhibition of Kv2.1 current amplitude by flotillin-1 was independently to the proteasome degradation.

Flotillins have additionally been reported to modulate the receptor proteins on the membrane by altering their intracellular trafficking to membrane (Solis et al., 2012). We thus investigated whether flotillin-1-induced inhibition of Kv2.1 current was due to a decrease in the amount of Kv2.1 proteins transported to membrane. The membrane and cytosolic protein fractions in control or flotillin-1 treated HEK-293 cells were isolated by a biotinylation assay, and Kv2.1 was then detected by immunobloting with an anti-Kv2.1 antibody (Fig. 2G). The membrane bound Na-K ATPase was used as a control for the membrane fraction, while GAPDH was used as a control for the cytosolic fraction. The results indicate that the membrane fraction of Kv2.1 was not significantly decreased with overexpression of flotillin-1 (100.31% ± 16.83% of the untreated, *n* = 5, *P* > 0.05). Moreover, knockdown of flotillin-1 by shRNA targeting flotillin-1 also did not alter the Kv2.1 levels in the membrane (94.91% ± 10.06% of the untreated, *n* = 5, *P* > 0.05). Taken together, these findings suggest that flotillin-1 inhibits Kv2.1 current amplitude, without affecting overall Kv2.1 expression, or its trafficking to membrane.

Besides freely diffusing across the plasma membrane, Kv2.1 was found to be concentrated in micron-sized clusters in neurons and HEK-293 cells (Kihira et al., 2010; O’Connell et al., 2010). We thus investigated whether overexpression of flotillin-1 affected the Kv2.1 clustering on the membrane using fluorescent imaging techniques. In this experiment, Kv2.1 α-subunit was transected into HEK-293 with or without overexpressing flotillin-1 and then labeled by a specific Kv2.1 antibody. The results obtained by fluorescent imaging show that the co-expression of flotillin-1 significantly increased Kv2.1 clustering on the membrane (Fig. 2H). Line scanning analysis clearly showed that co-expression of Kv2.1 and flotillin-1 resulted in more Kv2.1 clusters, when compared to expression of Kv2.1 alone (Fig. 2I). These results showed that the Kv2.1 current amplitude modulation through flotillin-1 was mainly through the Kv2.1 clustering on the membrane.

Flotillin/reggie are widely expressed proteins associated with non-caveolar rafts and are known to play important roles in the various cellular process (Langhorst et al., 2005; Stuermer, 2011). However, whether flotillin-1 can modulate ion channels on cell membranes has yet to be shown. Our study presents pivotal findings, showing that flotillin-1 can decrease *I*_K_ and Kv2.1 current amplitude, in native neurons and HEK-293 cells, and that flotillin-1 may mediate decreases in channel conductance by increasing Kv2.1 surface clustering. Our results also showed that it is highly likely that the overexpression of flotillin-1 increased the formation of nonconducting clusters of Kv2.1 channels, resulting in the decrease of Kv2.1 current amplitude. Due to the fact that this type of surface clustering at membrane is a specific property of Kv2.1 channels, the mechanism for flotillin-1-mediated Kv2.1 clustering and current inhibition may be unique for Kv2.1. However, further studies must be carried out to determine if these results can be extended to other channel proteins that also localize to non-caveolar lipid rafts.

Our results here unveil a novel role for flotillin-1, as a scaffolding protein affecting the regulation of membrane ion channel function. Since Kv2.1 channels mediate neuronal excitability, development, and migration, it is likely that flotillin-1 may also regulate neural excitability and development by modulation of these channels.

## Electronic supplementary material

Below is the link to the electronic supplementary material.

Supplementary material 1 (PDF 222 kb)
