# Supplementary material for: Flotillin-1 downregulates K+ current by directly coupling with Kv2.1 subunit
Source: Protein Cell. 2016 May 24;7(6):455–60. doi: 10.1007/s13238-016-0276-3 (PMC4887332; doi:10.1007/s13238-016-0276-3)
Supplement: Supplementary file 1 — Supplementary material 1 (PDF 222 kb) [file 13238_2016_276_MOESM1_ESM.pdf]

Supporting information

Flotillin-1 downregulates K^+ current by directly coupling with Kv2.1 subunit

Rui Liu*, Guang Yang*, Meng-Hua Zhou*, Yu He, Yan-Ai Mei# and Yu Ding#

School of Life Sciences, Institutes of Brain Science and State Key Laboratory of Medical Neurobiology, Fudan University, Shanghai 200438, China

Corresponding author E-mail: yamei@fudan.edu.cn, yuding@fudan.edu.cn

Supplementary Data

Materials and Methods

Primary cell culture

This study was carried out in strict accordance with the recommendations in the Guide for the Care and Use of Laboratory Animals of the National Institutes of Health. The protocol was approved by the Committee on the Ethics of Animal Experiments of the Fudan University (Permit Number: 20090614-001). All surgery was performed under sodium pentobarbital anesthesia, and all efforts were made to minimize suffering. CGNs were derived from the cerebellum of 7 day old Sprague-Dawley rat pups as described previously [1, 2]. Isolated cells were plated onto 35 mm Petri dishes coated with poly-L-lysine (1 μg/ml), at a density of 10^6 cells/ml. Cultured cells were incubated at 37°C under 5% CO_2, in DMEM supplemented with fetal calf serum (10%), insulin (5 μg/mL), KCl (25 mM), and 1% antibiotic- antimycotic solution. After culturing for 24 h, cytosine β-D-arabinofuranoside (5 μM) was added to the culture medium to inhibit the proliferation of non-neuronal cells. All experiments were performed using primary CGNs after 7-8 days in culture.

Molecular cloning and HEK-293 cells transfection

Total RNA was isolated from primary cultured rat cortical neurons, according to the manufacturer's instructions (Qiagen Mini RNeasy; QIAGEN, Valencia, CA). First-strand synthesis was performed using SuperScript II reverse transcriptase (Invitrogen, Carlsbad, CA). Amplification was performed with the following primer sets: Primer Kv2.1: forward, 5'-TGGCTCGAGATGCCGGCGGGCATG 3'(XhoI); reverse, 5'-ATACAGAATTCGGATACTCTGATCCCT 3'(EcoRI), GenBank NM_008420. Kv2.1 cDNA was ligated into pEGFPN1 by using XhoI and EcoRI restriction sites. Each gene was expressed with enhanced green fluorescent protein (eGFP) or red fluorescent protein (mCherry) fused to the N terminus, which was used as the marker to detect the channel when transfected into human embryonic kidney (HEK) 293 cells. The short hairpin RNA (shRNA) construct used to knockdown flotillin-1 expression was generated by inserting the designed flotillin-1 shRNA into *Hpa* I and *Xho* I site of pLentiLox 3.7 (pLL3.7) shRNA vector. All the constructs were verified by sequencing. The plasmids were extracted using a Qiagen plasmid midi kit (QIAGEN). The DNA concentration and purity were determined by measuring the absorbance at 260 and 280 nm. HEK-293 cells were transfected using the lipofectamine 2000 reagent (Invitrogen, Carlsbad, CA). The average transfection efficiency was >80%. Two days after transfection, HEK-293 cells with fluorescence were further analyzed.

Patch-clamp recordings

Whole-cell currents of granule neurons were recorded using a conventional patch-clamp technique. Prior to I_K current recording, the culture medium was replaced with a bath solution containing 145 mM NaCl, 2.5 mM KCl, 10 mM HEPES , 1 mM $MgCl_2$, 0.001 mM TTX, 5 mM 4-AP, and 10 mM glucose (pH adjusted to 7.4 with NaOH). Soft-glass recording pipettes were filled with an internal solution containing potassium 135 mM gluconate, 10 mM KCl, 10 mM HEPES, 1 mM $CaCl_2$, 1 mM $MgCl_2$, 10 mM EGTA, 1 mM ATP, and 0.1 mM GTP (pH adjusted to 7.2 with KOH). Cultured granule cells exhibiting similar morphological characteristics were selected for electrophysiological recordings, which were performed at room temperature. All currents were recorded using a multiclamp 700B amplifier (Axon Instruments, Foster City, CA, USA), operated in voltage-clamp mode. The current was digitally sampled at 100 μs and the current signals were filtered by a 10-KHz lowpass frequency filter. Data acquisition and analysis were performed with pClamp 10.2 software (Axon Instruments) and/or Origin 6.1 analysis software (Microcal Software, Northampton, MA, USA).

In the experiments examining the steady-state activation property of I_K, the membrane potential was held at –70 mV, and I_K was evoked by a series of 200 ms depolarizing pulses, increasing from a first pulse potential of –70 mV to +100 mV, in 10 mV steps, at 10 s intervals. The data was analyzed using the equation GK = I_K / (Vm-Vrev), where GK is the membrane K^+ conductance, Vm is the membrane potential, and Vrev is the reversal potential for K^+.

To study steady-state inactivation of I_K channels, currents were elicited using 10 s conditioning pre-pulses, from –90 mV to +10 mV, before a 400 ms test pulse of +10 mV. After normalizing each current amplitude to the maximal current amplitude obtained from the –90 mV pre-pulse, as a function of the conditioning pre-pulse potential, and fitting the normalization with the function $I_K/I_K\text{-max} = 1/(1+\exp[(Vm1/2–Vm)/k])$, we obtained an inactivation curve for I_K and calculated Vh_{50} (the voltage at which the current amplitude was half-inactivated).

Western blot analysis

Cells were lysed in HEPES-NP40 lysis buffer (20 mM HEPES, 150 mM NaCl, 0.5% NP-40, 10% glycerol, 2 mM EDTA, 100 μM Na_3VO_4, 50 mM NaF, pH 7.5, and 1% proteinase inhibitor cocktail) on ice for 30 min. After centrifugation, the supernatant was mixed with 2× sodium dodecyl sulfate loading buffer and boiled for 5 min. The proteins were separated on a 10% SDS-polyacrylamide gel and transferred to polyvinylidene difluoride membranes (Millipore, MA USA). The membrane was blocked in 10% nonfat milk and incubated at 4°C overnight, with mouse monoclonal antibody against Kv2.1 (1:1000; UC Davis, USA), mouse anti flotillin-1 monoclonal antibody (1:1000, BD Biosciences, USA) or mouse anti Na-K ATPase (1:1000; Sigma, USA). After extensively washing in TBST, the membrane was incubated with horseradish peroxidase-conjugated anti-mouse or anti-rabbit IgG (1:10,000) (KangChen Bio-Tech, China) for 2 h at room temperature. Proteins were visualized by chemiluminescent signals generated using a SuperSignal West Pico kit (Pierce,

USA), and detected by exposure to X-ray film or Chemidoc (XRS+, Bio-Rad Laboratories). Quantity One (Version 4.6.2, Bio-Rad Laboratories) was used for quantification and background subtraction of immunoblots.

Immunocytochemistry

Immunocytochemistry on cultured cells was carried out as described previously [3]. Briefly, the cells were fixed on glass slides for 15 min with 4% paraformaldehyde, washed twice for 5 min in PBS, blocked for 1 h in 1% BSA, incubated with primary antibody overnight at 4°C, washed 3 times for 5 times in PBS and then incubated with secondary antibody. We used antibodies to Kv2.1 (1:200; UC Davis, USA) or to flotillin-1 (1:200; Santa Cruz biotechnology, Inc) as primary antibodies, the cell nuclei were stained with DAPI (Invitrogen, Carlsbad, CA). We used goat antibodies to mouse or to rabbit (Biyuntian, China) at 1:500 as secondary antibodies.

Construction of Chimera Plasmids and the Bimolecular Fluorescence Complementation (BiFC) Analysis

We first constructed a series of chimera plasmids for the BiFC analysis. Each of these plasmids contain the sequencing encoding N-terminal 1-172 aa of superfolder GFP (sfGFP) or C-terminal 173–238 aa of sfGFP fused with the protein tested, either flotillin-1, Kv2.1 or flotillin-2. We made constructs of pFlot1-NsfGFP / pFlot1-CsfGFP, pKv2.1- CsfGFP / pKv2.1-NsfGFP, as well as control plasmids pNsfGFP / pCsfGFP (negative control), and pFlot2-CsfGFP / pFlot2-NsfGFP

(positive control). All DNA sequences of the constructs used for BiFC analysis was verified by restriction mapping and nucleotide sequencing.

HEK-293 cells were cultured in DMEM medium with 10% fetal bovine serum (FBS) including penicillin and streptomycin. For BiFC visualization, HEK-293 cells were cultured on coverslips in the confocal dishes, and transiently transfected with NsfGFP and CsfGFP chimera plasmids using Lipofectamine 2000 reagent (Invitrogen, Carlsbad, CA) according to the provider's instructions. After 24 h incubation at 37°C, the confocal dishes were directly examined with a Leica TCS SP5 II confocal laser scanning microscope. The in vivo interaction of the NsfGFP and CsfGFP tagged chimera proteins were detected by excitation at 450-490 nm and emission at 515 nm.

Biotinylation assay

The cell surface proteins were biotinylated according to the manufacturer's protocol as previously described [3]. Briefly, the cells were incubated with 0.25 mg/ml of Sulfo-NHS-SS-biotin (Thermo scientific, Rockford, USA) for 45 min and then blocked with 50 mM of Tris (pH 8.0) for 20 min at 4°C. The cells were lysed in HEPES-NP40 lysis buffer. Biotinylated proteins were pulled down using streptavidin agarose beads (Thermo scientific, Rockford, USA) overnight at 4°C and then washed 4 times with lysis buffer. The bound proteins were eluted with the sample buffer and then used for western blotting.

Co-immunoprecipitation (Co-IP)

The cells were harvested as described in the western blotting section. The

monoclonal antibody against Kv2.1 (NeuroMab, clone number K89/34) was incubated with dynabeads protein G (Invitrogen, Carlsbad, CA), then BS3 (Pierce, Rockford, IL) was added to a final concentration of 4mM at 4ºC. Two hours later, the quenching solution (1M Tris-HCl, pH 7.5) was added to stop the cross linking reaction. Then the Kv2.1 antibody cross-linked dynabeads was washed and incubated with the supernatants of transfected cells overnight at 4ºC. The dynabeads were washed 3 times and the binding proteins were finally eluted by 100mM triethylamine, resuspended in sample buffer and boiled for 5 min. Then western blot analysis was performed on the supernatants. The blots were then probed with either a mouse monoclonal antibody against flotillin-1 or Kv2.1 (1:2000 each, anti flotillin-1 from BD Biosciences and anti Kv2.1 from NeuroMab).

Line scan analysis

The plasmids encoding mCherry-Kv2.1 α-subunit and eGFP-flotillin-1 were co-transfected into HEK-293 cells while eGFP was co-transfected with mCherry-Kv2.1 as the negative control. The red channel image obtained by a Leica TCS SP5 II confocal laser scanning microscope was further analyzed by line scan analysis using ImageJ [4]. The fluorescence intensity was measured in 120 pixel lines.

Statistical analysis

Statistical analysis was performed using Student's *t*-test with non-paired comparisons, or paired comparisons when appropriate. Values are expressed as the

mean ± SEM, with n as the number of cells tested. A P value < 0.05 was used to denote a significant difference between groups. When multiple comparisons were made, data were analysed by a one-way ANOVA test.

Reference:

1. Zhou, M. H., Yang, G., Jiao, S., Hu, C. L. & Mei, Y. A. (2012) Cholesterol enhances neuron susceptibility to apoptotic stimuli via cAMP/PKA/CREB-dependent up-regulation of Kv2.1, *J Neurochem.* **120**, 502-14.
2. Gonzalez, B., Leroux, P., Lamacz, M., Bodenant, C., Balazs, R. & Vaudry, H. (1992) Somatostatin receptors are expressed by immature cerebellar granule cells: evidence for a direct inhibitory effect of somatostatin on neuroblast activity, *Proc Natl Acad Sci U S A.* **89**, 9627-31.
3. Yao, H., Zhou, K., Yan, D., Li, M. & Wang, Y. (2009) The Kv2.1 channels mediate neuronal apoptosis induced by excitotoxicity, *Journal of neurochemistry.* **108**, 909-19.
4. Kihira, Y., Hermanstyne, T. O. & Misonou, H. (2010) Formation of heteromeric Kv2 channels in mammalian brain neurons, *J Biol Chem.* **285**, 15048-55.

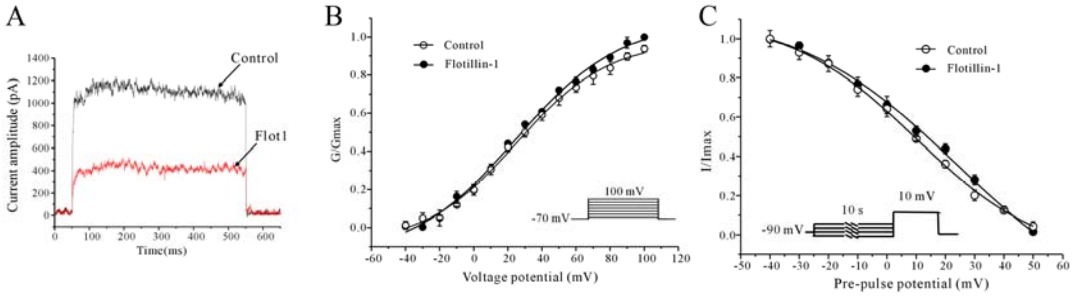

<u>Figure S1</u> The effects of overexpression of flotillin-1 on I_K amplitude, steady-state activation, steady-state inactivation properties in CGNs. (A) I_K evoked by a 200 ms depolarizing pulse from a holding potential from -50 to +40 mV. Separate current traces were obtained from cerebellar GCs before and after overexpression of flotillin-1. (B) Steady-state activation curves of I_K obtained by plotting the normalized conductance as a function of command potential, obtained from control and flotillin-1-treated groups. Data points shown as means ± SEM were fitted using the Boltzmann function. (C) Steady-state inactivation curves for I_K were obtained from control and flotillin-1-treated CGNs, using inactivation protocols.

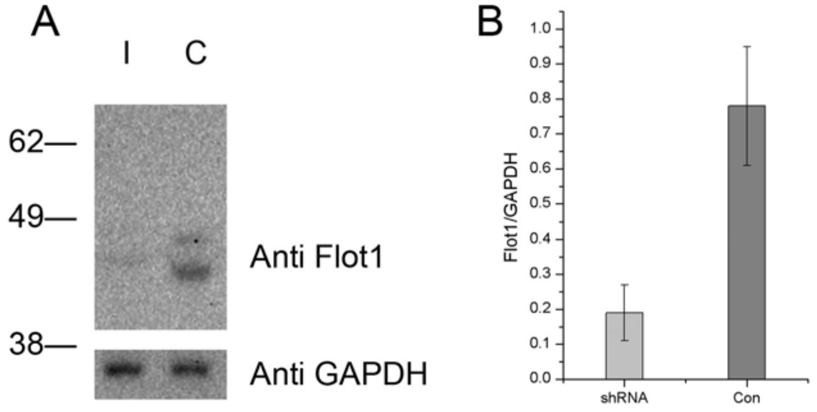

<u>Figure S2</u> Expression levels of flotillin-1 after shRNA transfection. A. The HEK-293 cells were transfected with the pLL3.7-flotillin-1 shRNA (I) or scramble shRNA control (C), then the expression level of flotillin-1 was visualized by Western blot and GAPDH was used as the internal control. B. The statistical analysis of flotillin-1 expression level after shRNA treatment (n=3). Data points are shown as mean ± SD.

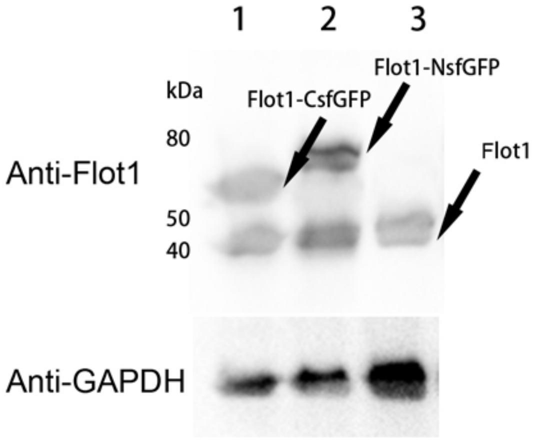

<u>Figure S3</u> **Expression levels of flotillin-1 after transfection.** The HEK-293 cells were transfected with the following: Lane 1 (Combination A: Flot1-CsfGFP and Kv2.1-NsfGFP), Lane 2 (Combination B: Flot1-NsfGFP and Kv2.1-CsfGFP) and Lane 3 (Untransfected cells as negative control). The expression of flotillin-1 was visualized by anti-flotillin-1 antibody. Our results showed that combination A's expression level of transfected Flot1-CsfGFP was 104% that of endogenous flotillin-1 (with total flotillin-1 normalized to 100%, consisting of 51.0% Flot1-CsfGFP and 49.0% of endogenous flotillin-1). Similarly, combination B's expression level of Flot1-NsfGFP was 111% that of endogenous flotillin-1 (with total flotillin-1 normalized to 100%, consisting of 52.6% of Flot1-NsfGFP and 47.4% of endogenous flotillin-1).
